# Safe surgical treatment of peripelvic renal cyst combined with renal calculi by percutaneous nephroscopy

**DOI:** 10.1002/ccr3.1302

**Published:** 2018-01-05

**Authors:** Bowei Yang, Jiongming Li, Jianhe Liu, Yuepan Bao, Prashant Mishra, Haixiang Guo, Pei Li, Yongming Jiang

**Affiliations:** ^1^ The 2nd affiliated hospital of Kunming medical university Kunming China; ^2^ Lan Cang hospital Pu Er China

**Keywords:** Percutaneous nephroscopy, peripelvic, renal cyst, treatment

## Abstract

We have tried to establish a safe and effective method to treat the peripelvic renal cyst combined with renal calculi. The key points are as follows: choose the appropriate target calyx; find the gap between cyst and renal pelvic; put a nephrostomy tube to stimulate the closing of cystic cavity.

## Introduction

Renal cyst is a common disease in urology. An autopsy study showed that half of over 50‐year‐old patients had at least one renal cyst 1. Most cysts are asymptomatic, but some can cause pain, obstruction, hematuria, and infection which depend on the size and location of cysts 2. Renal cysts could be diagnosed by ultrasonography or other radiologic examination. Usually, simple renal cyst is anechoic, with a hairline thin wall in ultrasonography. By computer tomography (CT) scan, renal cyst measures as water density in Hounsfield units and does not enhance with intravenous contrast agent. In 2005, Bosniak characterized renal cyst into five classes called “the Bosniak classification of renal cysts,” which is the most useful and widely employed method. It used the imaging characteristics (such as calcification, solid component, and density) to separate cysts into five classes. The higher class might have a high incidence of malignancy and needed for excision or ablation 3. In clinic, they also separated the renal cyst by its positions, for example, perirenal cyst, peripelvic renal cyst, and intrarenal cyst. Usually, the perirenal cyst is asymptomatic until it becomes large enough to cause pain or shows signs of compression, but the peripelvic renal cyst can cause renal pelvic compression at early stage which may cause pain and obstruction.

Treatment modalities for different location's renal cysts are varied. In broad sense, it can be separated into two parts: through percutaneous and surgical (either laparoscopic or open). Percutaneous aspiration or marsupialization is a safe and cost‐effective to treat the simple and perirenal cysts 4. Surgical unroofing (either laparoscopic or open) is also a safe and effective method, and it requires general anesthesia 5. These methods are fit for the perirenal cysts, because we can find these kinds of cysts at renal surface, and it is less likely to cause damage to renal parenchyma during treatment. But peripelvic renal cysts are totally different because it is completely intrarenal, which make it very difficult and challenging to treat through laparoscopic or open surgery. Moreover, these cysts are very close to the renal vessels and renal pelvis. So we tried to treat such kind of cyst by percutaneous nephroscopy (PCN). PCN can treat these kinds of cysts intrarenal, preventing renal vessel damage, and can choose suitable way to unroof the cyst as wide as possible. After unroofing the cyst, the cavity will be closed and disappear due to inflammatory reactions of urine.

## Case History

The patient was 65 years old, obese female, farmer by profession suffering from right lumbago for 3 months. Three months ago, she had sudden right lower back pain for which she visited to orthopedic clinic and was diagnosed as mechanical pain and prescribed with painkiller, but she just had temporary relief with painkiller and her symptoms still persist. So she went to the community hospital for further consultation and was ordered abdominal ultrasound examination and found to have a renal stone and mild hydronephrosis in her right kidney. Then, she decided to come to our hospital for further treatment.

## Clinical Findings

She had no history of surgery or any chronic disease, no significant family history of genetic disease and psychosocial disorder. On physical examination, she had right renal tenderness on percussion, blood pressure was 145/90 mmHg, and the rest was unremarkable.

**Figure 1:**
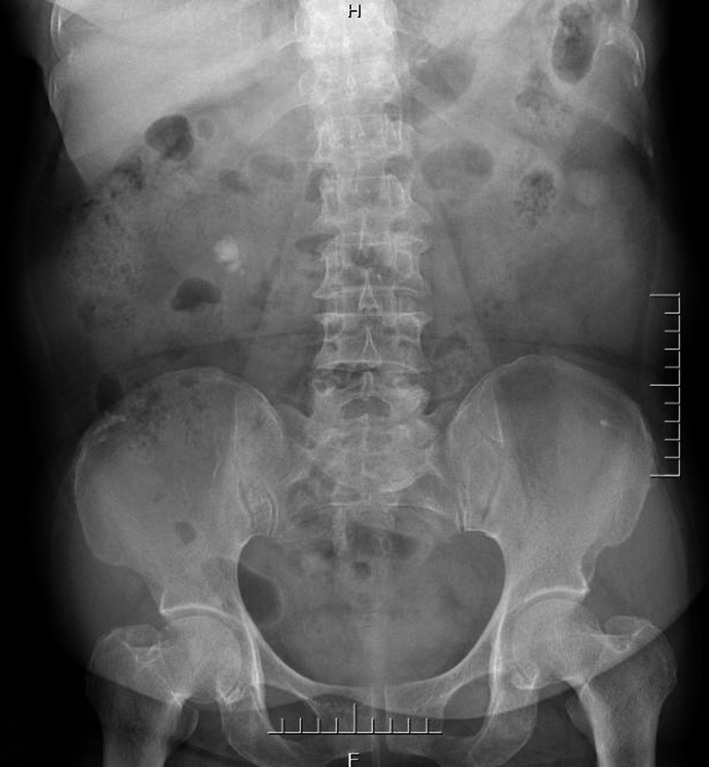
Preoperation KUB.

**Figure 2:**
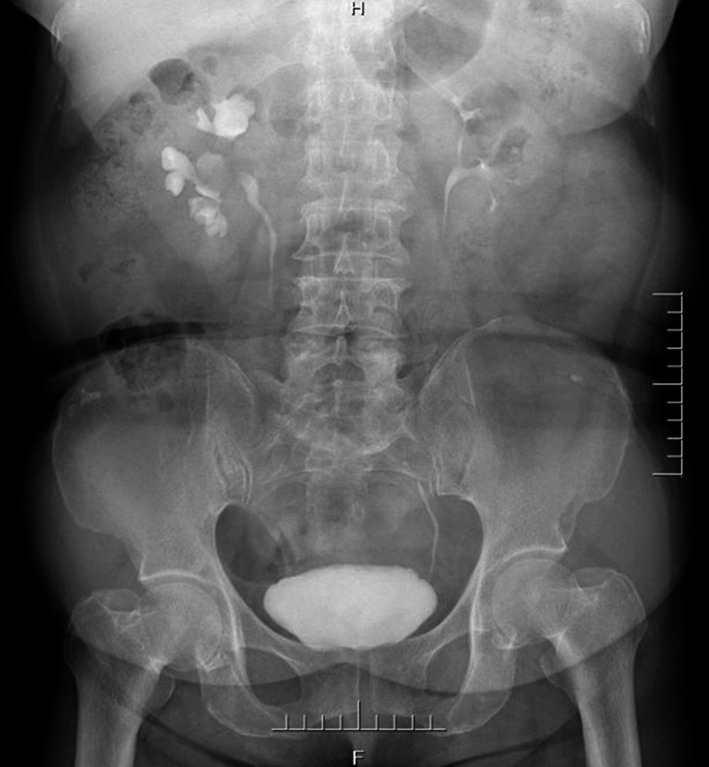
Preoperation IVU.

**Figure 3:**
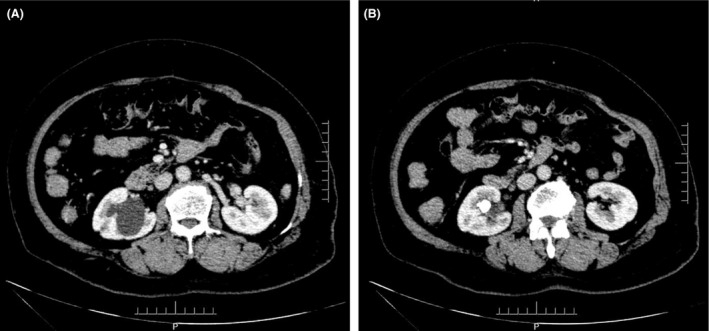
(A and B) Preoperation CT.

**Figure 4:**
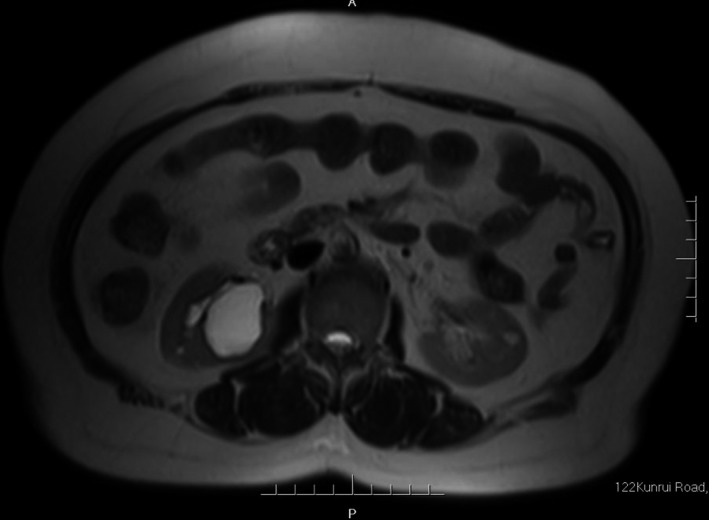
Preoperation MRI.

**Figure 5:**
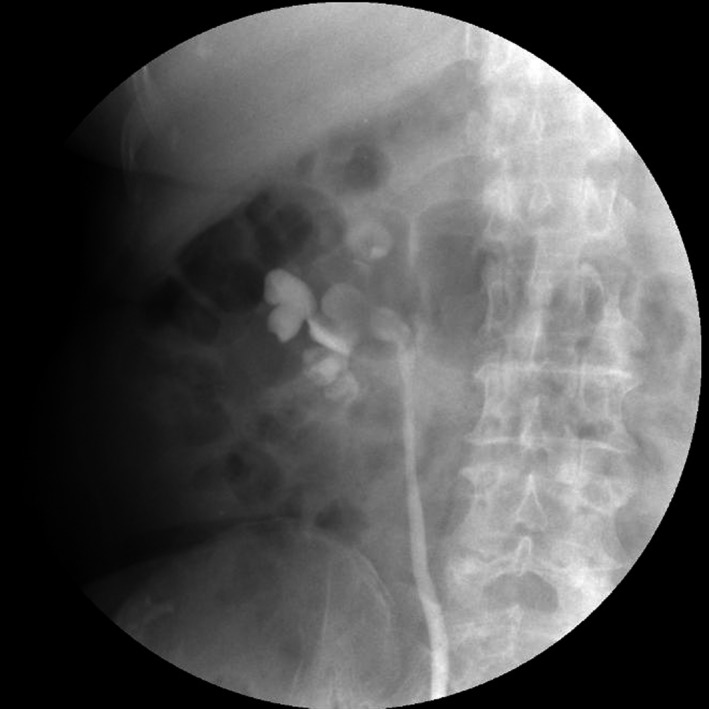
Preoperation retrograde pyelography.

Complete blood count (CBC) was normal. The urinalysis showed WBC (1+), renal function, and coagulation profiles were WNL, but her lipid profile showed high cholesterol level. On radiological examination, KUB found to have 2 cm calculus in right kidney (Fig. 1). Intravenous pyelogram (IVP) (Fig. 2) showed a vallecula inside the right kidney, which was compressing the pelvis causing mild hydronephrosis. Contrast‐enhanced CT (CECT) scan (Fig. 3A and B) showed a hypodense, nonenhancing lesion inside the right kidney, which was compressing the right renal pelvis and middle calyx causing mild hydronephrosis. There was a 2‐cm calculus in right lower calyx next to the cyst. In order to evaluate the cyst and confirm the diagnosis, an MRI (Fig. 4) and retrograde pyelography (Fig. 5) were carried out. MRI showed a hypointense lesion inside the renal sinus, causing right renal pelvis compression and retrograde pyelography found a significant compression in right renal pelvis.

## Diagnostic Focus and Assessment

According to the patient's history and investigations, first the diagnosis of this patient was right renal calculus measuring 2 cm with mild hydronephrosis, but this hydronephrosis was not caused by the renal calculus, and more likely there was a mass which was compressing the renal pelvis causing mild hydronephrosis. So in order to make the confirmatory diagnosis for this mass, a contrast CT scan, MRI, and retrograde pyelography were performed. Contrast CT scan found a low‐density peripelvic lesion, which was completely inside the kidney with no enhancement, and the lesion compressed the right renal pelvis causing mild hydronephrosis. MRI also showed a hypointense peripelvic lesion inside the kidney, causing right renal pelvic compression. Retrograde pyelography also showed there is a significant compression in right renal pelvis. Following all these investigation, the diagnosis of this mass was a peripelvic renal cyst completely inside the kidney compressing renal pelvis and causing hydronephrosis.

## Therapeutic Focus and Assessment

**Figure 6:**
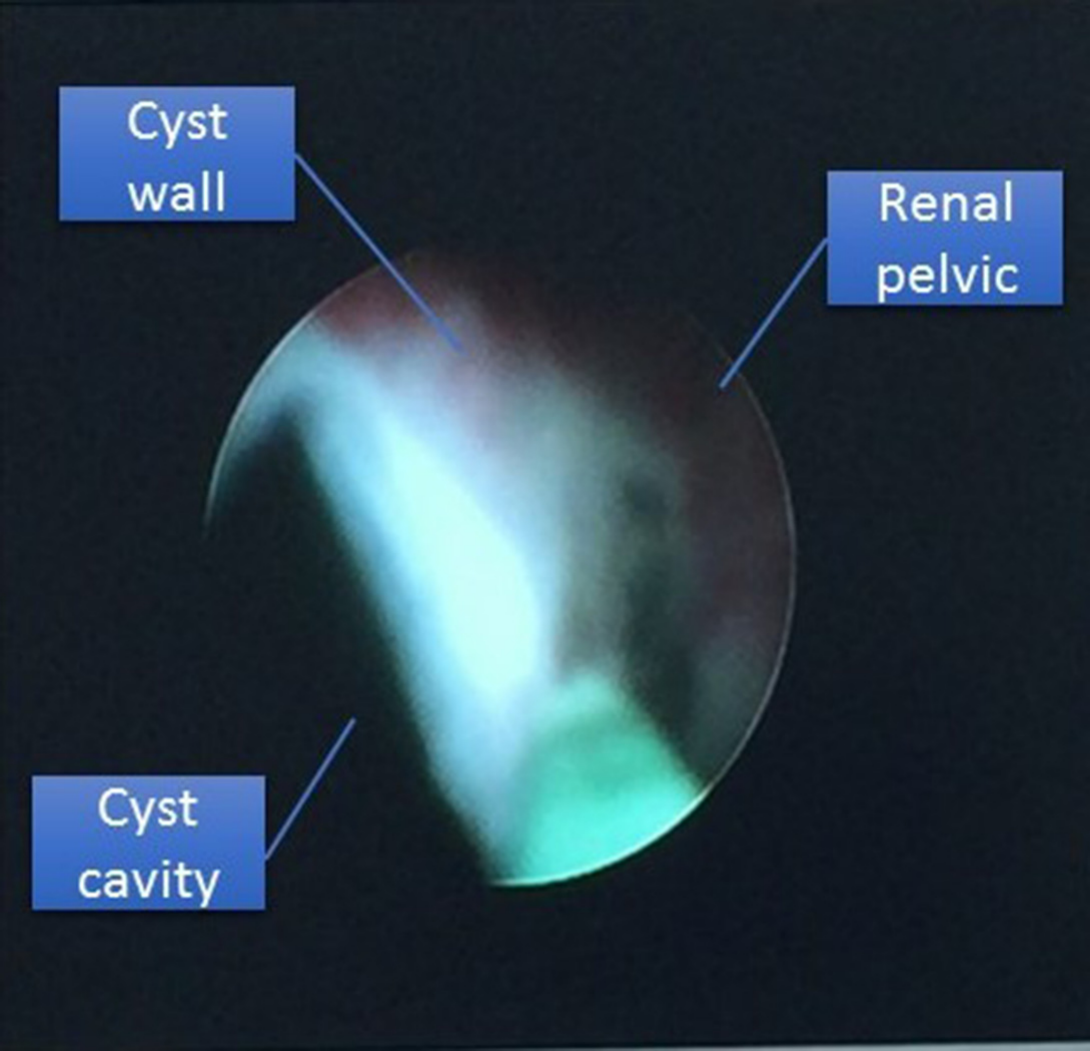
Gap between renal pelvic and cyst wall by ureteroscope.

The patient had 2 cm renal calculus and peripelvic renal cyst. In order to treat these two diseases at the same time, we choose PCN as the first choice because treating the cyst from surface of the kidney is very difficult in such patient with peripelvic cyst. By PCN, the stone was cleared first; then, the cyst was unroofed intrarenally. Under general anesthesia, retrograde ureteral catheterization was done in lithotomy position. Then, patient was kept in left lateral position due to the obesity. About 500 mL normal saline was infused through the urethral catheter to create the hydronephrosis. Meanwhile sterilization and draping was done. Target calyx was middle calyx, under ultrasound guidance middle calyx was punctured, avoiding the cyst to get punctured. After successful puncture and insertion of guide wire, step‐by‐step dilation was performed and 18 Fr working channel was used. 8 ‐ 8.9Fr nephroscope was used to find the calculus in the lower calyx, and the stone was cleared by holmium laser and flushed. Then, the scope was removed, but the channel was intact. Ultrasound was used again to observe the cyst, and through the same channel, puncture needle was inserted toward the cyst, and channel was extended into the cyst. This channel had to meet three conditions: (1) The punctured point of cyst wall should be thin enough. (2) Preventing the vessel damage (especially the renal artery and vein). (3) Puncture the cyst through the channel that was used to break the stone. After puncture, cystic fluid was sucked and cyst shrinks. As the cyst was already shrunk, a gap between renal pelvic and cyst wall could be easily visible by nephroscope (Fig. 6). Under nephroscopic vision, this gap was isolated as large as possible until adhesion. In order to enlarge the channel from renal pelvic to cyst cavity, about one‐third of the cyst wall was incised by holmium laser; then, part of the renal pelvis was incised which was close to the cyst wall. After that, the double J stent was inserted as usual and checked for any bleeding or stone fragments, and once assured, nephrostomy tube (18Fr) was placed with side hole into the cyst cavity so that it could preserve the tunnel from cyst to renal pelvis and drains the urine from renal pelvic to cyst in order to stimulate the cystic cavity closure. The operation time was about 2 h, and intraoperative bleeding was around 50 mL.

## Follow‐up and Out Comes

**Figure 7:**
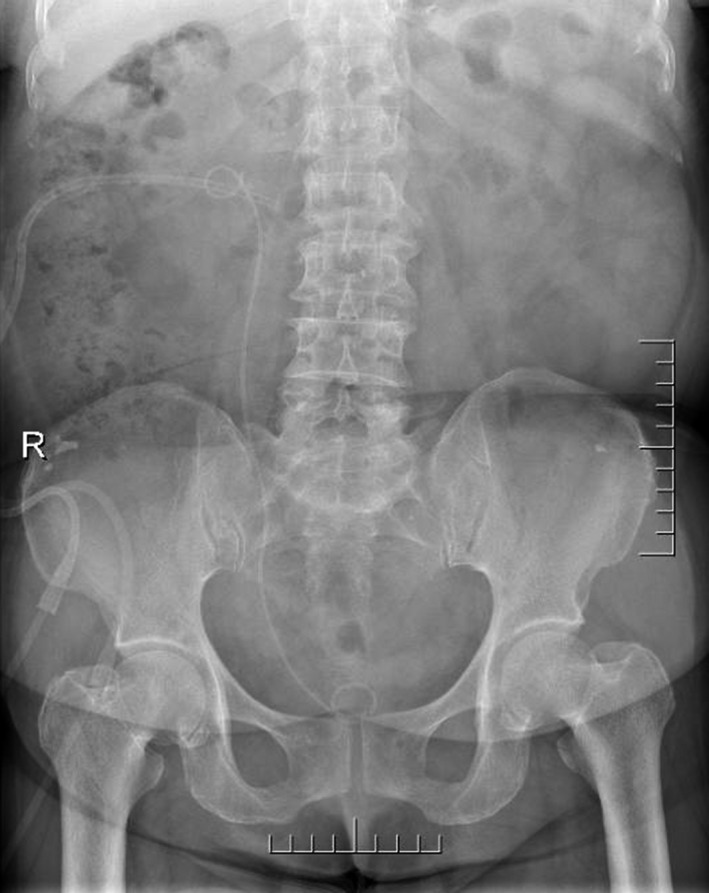
Postoperation KUB.

**Figure 8:**
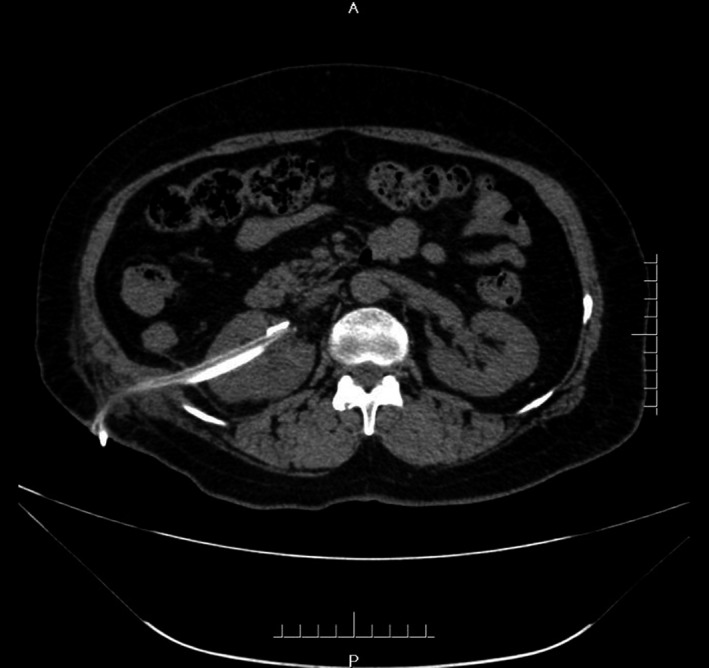
Postoperation CT.

**Figure 9:**
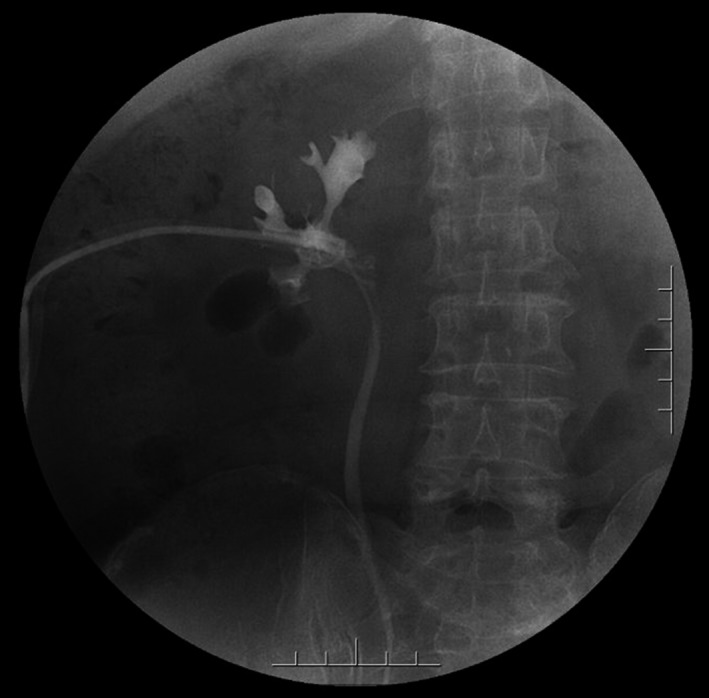
Postoperation Anterograde pyelography.

**Figure 10:**
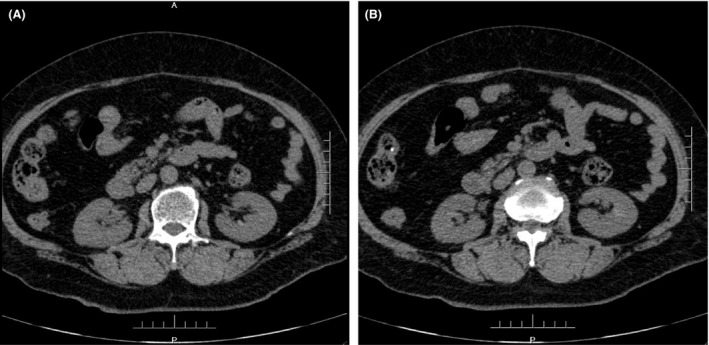
(A and B) Postoperation CT (1 month later).

This patient had no postoperative infection, bleeding , or severe pain. One week after surgery, KUB was carried out (Fig. 7), which showed the double J stent and the nephrostomy tube are in position, followed by CT scan (Fig. 8), which showed that the nephrostomy tube was already in the renal sinus where the peripelvic cyst was exist, and the double J stent was in position. No recurrent cyst and hydronephrosis in the right kidney was noted. Two weeks after operation, anterograde pyelography through the nephrostomy tube was performed (Fig. 9) which did not show hydronephrosis, and compression in the right kidney and tube was still inside the renal sinus, cyst cavity was not noticed and the shape of the calyx had become normal. After this, the double J stent was removed by ureteroscopy, and patient had no discomfort after stent removal. One month after the operation, the nephrostomy tube was removed and CT scan was performed (Fig. 10A and B), which showed that the peripelvic renal cyst had disappeared, and compared with the preoperation CT, and all calyxes were normal in shape and size. No hydronephrosis was noted. The renal function was normal, and patient had no pain.

## Discussion

Peripelvic renal cyst is the one which is originating from renal sinus 6. This kind of cyst is different from the one, which is originating from the renal cortex. Usually, the renal cyst is a perirenal one, but peripelvic renal cyst is mostly inside the kidney or renal sinus. So peripelvic renal cyst is difficult to treat from the renal surface. As we know, obstruction is one of the risk factors of renal stone formation. In this case, the peripelvic renal cyst has caused hydronephrosis, so this may be the reason that promotes the renal stone formation in this patient.

Recently, there have been some methods that have tried to treat the peripelvic renal cyst from intrarenal. Busato Jr W F S et. al. have reported to use a rollerball electrode to treat renal cyst by inspecting and cauterizing the inter cyst surface through a 26Fr percutaneous channel; this method has been shown to be a safe, minimally invasive, and effective technique for the management of large symptomatic renal cysts and is associated with high success rates and low complication rates in long‐term follow‐up 7. Also, some other methods have been reported using the retrograde ureteroscope to expand or fenestrate the renal cyst convex thin walls by holmium laser and make them communicate with the pelvis 8. In China, Shao Z Q et. al. have reported the treatment of peripelvic renal cyst by percutaneous intrarenal marsupialization. They reported the use of a 20.8 F nephroscope, and an incision made at the avascular area of the cystic wall by the 2 *μ*m laser to achieve intrarenal marsupialization. It is means: then kept one or two 6F double J stents in the ureter, and put the proximal end of these stents into the cyst cavity for 2‐3 months 9.

In this case, the patient had perirenal renal cyst and renal calculus. We tried to treat the cyst and the calculus simultaneously, so percutaneous aspiration or marsupialization was not very suitable because we could not treat the calculus by these methods, and it is difficult to puncture the cyst directly by ultrasound from renal surface directly. The PCN was suitable for this kind of patient, because we could use PCN to treat the calculus first, and then from inside of the renal, we could easily find the cyst and puncture it directly. Compared with other similar method, there are three advantages for this method: (1) Punctured the cyst through the same channel that we used for treating stone guided by ultrasound so that we could minimize the trauma caused by surgery. (2) Isolated the cyst wall and renal pelvic under ureteroscopic vision so that we can be sure that the vessel in renal sinus would not be damaged, and we can cut the cyst wall as large as possible. (3) After surgery put a nephrostomy tube (with side hole) into the peripelvic renal cyst, through this tube the urine could be drained from renal pelvic to renal sinus, which can stimulate the inflammation in renal sinus and accelerate the closing of this peripelvic renal cyst.

From this case, we have tried to establish a safe and effective method to treat the peripelvic renal cyst, especially the one combined with renal calculi. The key points for this method are as follows: (1) Choose the target calyx, which can treat the stone and cyst simultaneously. (2) Find the gap between cyst and renal pelvic, isolated under direct vision. (3) Put a nephrostomy tube with side hole into the cystic cavity to stimulate the closing of cystic cavity.

## Conflict of Interest

None declared.

## Authorship

BY: paper writing; chief doctor of this patient. JL: Operation director. JL: Operation director. YB: Video recording, data collection. PM: language modify. HG: patient follow‐up; data collection. PL: operation assistant; data collection. YJ: main operator; paper writing.
